# Nurses’ Adherence to the Portuguese Standard to Prevent Catheter-Associated Urinary Tract Infections (CAUTIs): An Observational Study

**DOI:** 10.3390/nursrep13040120

**Published:** 2023-10-10

**Authors:** Filipe Paiva-Santos, Paulo Santos-Costa, Celeste Bastos, João Graveto

**Affiliations:** 1 Health Sciences Research Unit: Nursing (UICISA: E), Nursing School of Coimbra (ESEnfC), 3000-232 Coimbra, Portugal; paulocosta@esenfc.pt (P.S.-C.); jgraveto@esenfc.pt (J.G.); 2 School of Medicine and Biomedical Sciences (ICBAS), University of Porto, 4050-313 Porto, Portugal; 3 Nursing School of Porto, 4200-072 Porto, Portugal; cbastos@esenf.pt

**Keywords:** nurses, professional practices, practice guidelines as topic, urinary catheters, cross-infection

## Abstract

Urinary tract infections are among the most prevalent types of healthcare-associated infections (HAIs) in hospitals and nursing homes, and they are primarily a result of unnecessary catheter usage and inadequate care. In Portugal, epidemiological data indicate that catheter-associated urinary tract infections (CAUTIs) remain widespread in clinical settings, resulting in increased morbidity and mortality rates among vulnerable populations. This study aimed to assess urinary catheter use in an oncology ward in Portugal and to evaluate nurses’ adherence to the government-endorsed standards for preventing CAUTIs. An observational study was conducted over a four-month period with daily assessments of nurses’ practices during urinary catheter insertion and maintenance using a government-endorsed auditing tool. Data were collected through on-site observations and nurses’ feedback. The findings revealed a urinary catheter utilization rate of 17.99%. However, there was a lack of complete adherence to government-endorsed standards among oncology nurses (0%). These results indicate that current practices lack evidence-based standardization. Therefore, there is a need to develop and implement quality improvement initiatives to enhance patient safety and experiences.

## 1. Introduction

Healthcare-associated infections (HAIs) are among the most dangerous threats in modern medicine. According to the latest HAI prevalence survey by the European Centre for Disease Prevention and Control, 9.1% of hospital patients in Portugal had experienced at least one HAI [1].

Urinary tract infections are among the most frequent types of HAIs in hospitals [2], with 43–56% of these infections associated with urinary catheter use [3]. Although urinary tract infections are considered to be less harmful than other HAIs, the high prevalence of urinary catheterization in healthcare settings has resulted in a significant burden of infections, complications, and deaths related to catheter-associated urinary tract infections (CAUTIs). Bacteriuria caused by indwelling catheters often leads to inappropriate antimicrobial use, while urinary drainage systems can serve as reservoirs for transmissible, multidrug-resistant (MDR) bacteria [4].

It is estimated that 19–26% of hospitalized patients undergo urinary catheterization during their hospital stay [5,6,7]. However, up to 40% of these procedures may be inappropriate [8]. Approximately 10% of patients with urinary catheters develop CAUTIs, which thus poses a significant mortality risk to vulnerable populations [9]. Patients with cancer are a particularly vulnerable group because immunosuppressive chemotherapy is a risk factor for the development of CAUTIs [4].

In Portugal, a study conducted at an internal medicine ward found 14.5 infections per 1000 urinary catheter use days and 71% of CAUTIs in patients who did not meet the criteria for urinary catheterization [6]. To address this issue, in 2015, the Portuguese Directorate General of Health developed a clinical standard, based on the bundle concept [10], including a set of interventions for all patients with urinary catheters. Although this standard was expected to be implemented in healthcare organizations throughout the country, its adherence rate is currently unknown, which undermines any potential systemic efforts to improve care delivery [11].

This challenge is particularly relevant to Portuguese nurses, since, as in other countries, urinary catheterization is considered an interdependent intervention. Although urinary catheterization is typically prescribed by physicians, nurses are responsible for catheter insertion, maintenance, and surveillance [12]. Therefore, nurses are expected to keep up to date on the latest evidence-based practices to create safer healthcare experiences.

This study aimed to assess the prevalence of urinary catheter use in an oncology ward in Portugal and to evaluate nurses’ adherence to the government-endorsed standards to prevent CAUTIs.

## 2. Materials and Methods

### 2.1. Study Design

A quantitative, observational, and prospective study [13] was conducted to analyze nurses’ practices during catheter insertion and maintenance. Findings are reported according to the Strengthening the Reporting of Observational Studies in Epidemiology (STROBE) guidelines [14].

### 2.2. Setting

This study was carried out in a department of a cancer hospital in Portugal between July and October 2021. This department admits patients who require symptom management due to chemotherapy treatment or disease progression, as well as patients undergoing end-of-life care.

Participants were recruited during the month of June 2021. Eligible nurses were invited to participate in a study presentation session during their normal working hours. This session lasted 30 min, during which participants were informed about the relevance and objectives of the study and confidentiality was ensured. At the end of the presentation, nurses had time to clarify any doubts regarding their participation.

### 2.3. Participants

Inclusion criteria required that nurses had at least 6 months of experience in inpatient settings. Nurses who were in their induction periods were not included in the study.

### 2.4. Data Sources

Data were collected using two approaches. Firstly, in collaboration with the nurse manager of the department, a checklist was scored daily to calculate the rate of urinary catheter use. At 08:00 a.m., the ward manager recorded the number of patients with indwelling catheters, as well as the total number of active ward admissions.

Secondly, the lead researcher visited the ward from 08:00 a.m. to 16:30 p.m. and identified, with the aid of the nursing team, all patients with indwelling urinary catheters, or those who would require one. The lead researcher observed nurses’ practices during catheter insertion (criteria 1 and 2) and maintenance (criteria 3, 4, 5, and 6) using the government-endorsed auditing tool (i.e., standard number 19/2015 [11]), as shown in Table 1.

Criteria 1 and 2 covered interventions related to urinary catheter insertion, whereas criteria 3–6 covered the interventions related to catheter maintenance. Aside from the auditing tool, the lead researcher took field notes to support the post hoc assessment of each item.

### 2.5. Bias

Given the need for direct observation of practices, data were collected by the same investigator to minimize the potential for bias.

### 2.6. Study Size

As part of an action research project, data were collected in a single department of a hospital that had implemented stringent measures in response to the SARS-CoV-2 pandemic. Considering these circumstances, the research team opted to collect data for a maximum of 4 months. During this period, a minimum of 40% of catheter days were observed.

### 2.7. Statistical Methods

Quantitative data were analyzed using SPSS Statistics^®^ (version 24, IBM SPSS; Chicago, IL, USA), with frequencies and percentages as descriptive statistics. Bundle adherence was measured using the guidelines set forth by the Portuguese Directorate General of Health. All care episodes in which reliable data were not available for at least one of the bundle’s sub-criteria were excluded from this study to avoid bias.

## 3. Results

Data were collected between July and October 2021. All nurses (*n* = 35) agreed to participate in this study. The sample consisted mostly of women nurses (*n* = 32), with a mean age of 39.17 years (range: 26–64 years) and an average of 16.3 years of nursing experience (range: 3–40), of which 8.03 years (range: 1–27 years) in the inpatient service where this study was conducted. The vast majority (*n* = 31) had a bachelor’s degree in nursing, and one nurse had a master’s degree in nursing. Four nurses were specialists: two nurses in rehabilitation nursing, and two in medical-surgical nursing (one of them with management responsibilities).

The catheter utilization rate (catheter days per 100 patient days) was 17.99%, based on 2690 days of hospitalization and 484 days of catheter use.

Throughout the data collection period, the research team documented 9 episodes of urinary catheter insertion and 197 episodes of maintenance care.

Adherence to sub-criteria related to urinary catheter insertion care ranged from 66.7% to 100%, while adherence to sub-criteria related to maintenance care ranged from 1.5% to 100%. Given that it was impractical to observe more than one nurse at a time, some sub-criteria were not always observed. Detailed information about the recorded care practices can be found in Table 2.

### 3.1. Bundle Adherence: Catheter Insertion

Nine urinary catheter insertions were observed. All nurses reported having assessed the possibility of avoiding catheterization; 77.8% recorded the reasons for urinary catheterization; 66.7% used aseptic technique during catheter insertion. The rate of adherence to interventions related to the insertion of urinary catheters (criteria 1 and 2) was 55.56% (Table 3).

Although the reasons for urinary catheter insertion were not always documented in patients’ clinical records, the nurses provided some of them verbally: treatment for microorganisms identified in urine samples (Code 2); indication by physicians (Code 5 and Code 48); diuresis control for undergoing end-of-life care (Code 22 and Code 34); need for bed rest due to neutropenia, thrombocytopenia, and asthenia (Code 27); erythema and fibrin lesions in the genital area caused by incontinence pads (Code 33); suspected urinary infection (Code 37); requirement for strict diuresis control among critically ill patients (Code 47).

Urinary catheterization was performed by a single nurse (*n* = 3), two nurses (*n* = 3), or a nurse and a healthcare assistant (*n* = 3). In seven of the nine observed cases (77.8%), nurses used a urinary catheterization kit containing sterilized materials, such as a pair of Kocher forceps, one dissecting clamp, one small bowl, five pieces of non-woven gauze, and one sterile drape. Nonetheless, in seven of the nine observations (77.8%), nurses had to stop the procedure and collect additional material, such as another urinary catheter, urine collector bag, a bowl for genital hygiene, a towel for post-hygiene care, saline solution for cleaning the urethral meatus, gloves for genital hygiene, support for the urine collection bag, underpad, or a syringe to inflate the balloon with bi-distilled water.

### 3.2. Bundle Adherence: Catheter Maintenance

While a total of 197 episodes of maintenance care (criteria 3, 4, 5, and 6) were recorded, bundle adherence was calculated for episodes where data were complete across all sub-criteria within the bundle. Thus, bundle adherence related to maintenance care was calculated for 51 episodes, as detailed in Table 4. The rate of adherence to interventions related to the maintenance of urinary catheters was 0%.

## 4. Discussion

Evidence-informed practices in nursing are critical in ensuring quality standards during urinary catheterization. This is because they use the latest and most reliable research evidence to inform clinical decision making and enhance patient care. Nurses strive to deliver secure, efficient, and effective care while encouraging patient autonomy and involvement in their own care. Although evidence-based nursing care can enhance patient outcomes, reduce healthcare costs, and promote ongoing learning and development within the healthcare system, our research indicates that current care practices for patients with cancer who require urinary catheters are not standardized or updated in line with the latest evidence available.

The number of urinary catheters per 100 hospital days in this study was 17.99, higher than in other studies reporting this outcome [1,8,15].

Our analysis revealed that unstandardized aseptic techniques significantly contributed to the low overall bundle adherence. Specifically, our findings suggest that one in three patients (33.3%) undergoing catheter insertion may be at risk of experiencing an unsafe procedure because of the potential for bacteria to enter the urinary tract via the periurethral area, migrating from the catheter tubing into the bladder (endogenous infection), cross-contamination via the nurses’ hands, or direct contact with unsterile surfaces (exogenous infection). Previous research has shown that catheterized patients are at risk of acquiring new microorganisms at a rate of 3%–7% per day. This risk is further increased by the occurrence of biofilms on the surface of the catheter, which can reduce the effectiveness of antibiotics [3]. While the morbidity and mortality rates associated with CAUTIs are relatively low compared to those associated with other HAIs, patients with cancer often experience compromised immunity, and a high frequency of urinary catheter use, which are risk factors for these infections [4].

In addition to the lack of systematic documentation of the reasons for catheter insertion, the reasons nurses provided verbally were not in line with international guidelines. Examples of valid reasons for urinary catheter insertion include the following [16]: acute urinary retention; bladder outlet obstruction; need for accurate urinary output measurements in critically ill patients; perioperative use for some surgical procedures; healing of open sacral or perineal wounds in incontinent patients; need for prolonged immobilization; to improve comfort for those undergoing end-of-life care.

Another aspect that requires further improvement is the lack of hand hygiene and the use of personal protective equipment (PPE) during catheter insertion and maintenance. In this study, approximately one in three nurses did not perform hand hygiene before patient contact and care delivery (69.6%), which increased the risk of exogenous infection. These results are aligned with those of a previous systematic review that found an overall adherence rate of 60%–70% across different clinical settings [17].

Moreover, PPE use was not consistent throughout the observations, particularly the use of disposable aprons (65.2%). Phan and colleagues [18] found similar results, reporting an overall adherence to wearing the PPE specified for each isolation category of 60%. According to the authors, 90% of observed doffing was incorrect, based on the doffing sequence, doffing technique, or use of appropriate PPE. Dobrina and colleagues also found an improvement in nurses’ appropriate use of gowns and aprons before and after the COVID-19 pandemic, although the reported adherence rates varied between 45.33% and 65.67% [19].

Some authors have pointed out that health professionals’ adherence to hand hygiene and PPE use is a dynamic phenomenon determined by self-regulatory and social factors. Although our study did not explore the reasons for non-adherence, previous studies have found that health professionals’ beliefs about consequences, individual decision processes, knowledge of current guidelines, and environmental and context resources are the main reasons for non-adherence [20,21].

The prevalence of urinary catheter securement was significantly lower than expected (3.6%) in our study. However, low adherence to this practice has been documented in other studies [22]. These findings are concerning because inappropriate securement of urinary catheters can have a significant impact on the physical and psychological wellbeing of patients. Repeated, unintentional movement of the catheter can cause inflammation and irritation of the urethra, increasing the risk of bacterial migration and infection [23] and severe trauma to the patient’s penile or labial tissues (cleaving), urethra, and bladder neck. It can also lead to catheter bypassing and accidental dislodging, increasing the likelihood of unnecessary catheter replacement.

Although current evidence suggests involving patients and families in their care to reduce these infections [24,25], our research found that only a small proportion of patients (1.5%) had such an opportunity. Prior studies on patient and family engagement initiatives included teaching and training them about urinary catheter use, the risks associated with indwelling urinary catheters, how to recognize the signs of CAUTIs, periurethral skin care (e.g., cleaning the insertion site, hand hygiene, bathing recommendations), and urinary catheter maintenance (e.g., keeping a closed system, proper bag placement, bag changing/emptying procedures) [26,27]. A previous systematic review by Mangal and colleagues found that patient and family engagement interventions were associated with non-statistically significant improvements in CAUTI incidence rates, which may be explained by the lack of agreement between different authors on CAUTI-related outcomes and incidence metrics. Nonetheless, these interventions have been shown to enhance the care experience of patients and their families, resulting in a significant improvement in pre- and postoperative anxiety scores, patient satisfaction, patient/caregiver self-efficacy and knowledge, and quality of life. These authors also emphasized the role of these interventions in increasing patient satisfaction and trust in nursing care [26].

Lastly, inadequate nursing documentation can compromise the quality of patient care. Our study revealed that although most nurses recorded the reasons for catheter insertion (77.8%), only a few documented the reasons for keeping the catheter in place (7.6%). This disregard for nursing-sensitive outcomes related to catheterization suggests a failure to uphold the nursing process, which is a systematic, problem-solving approach that enables nurses to make clinical decisions and advocate for quality care. Neglecting autonomous interventions, such as the early identification of CAUTI symptoms and timely catheter removal, undermines nurses’ role as patient advocates. Poor nursing documentation practices can lead to fragmented care delivery, unmet patient needs, and adverse events. Failure to report the necessity of urinary catheterization can result in unnecessary catheterization days and increase the risk of significant complications such as CAUTIs.

Our study reveals that the rate of adherence to the maintenance care bundle was 0%, which suggests that the care provided is of low quality, mainly due to low adherence to patient education, catheter securement, and documentation of the reason for maintaining the catheter. Each of these interventions is part of a set of interventions that constitute three criteria. A bundle consists of 3–5 interventions that must be performed simultaneously to improve a given outcome [10], and there is a consensus that the use of bundles improves patient outcomes related to HAIs [28,29]. However, the Portuguese bundle includes several interventions in each criterion, which makes it difficult to increase bundle adherence. For example, the catheter maintenance bundle has 4 criteria with 12 interventions, which can be highly demotivating for professionals.

### Limitations and Considerations for Future Research

Our results should be interpreted with caution due to a number of limitations. Specifically, the cross-sectional design precluded conclusions on causality. Nonetheless, this study represents the first investigation of nurses’ adherence to the current government-endorsed bundle, and thus it is likely to generate interest among other health professionals, researchers, and managers in various clinical settings in Portugal. The dissemination of these findings may inspire others to replicate the study in other settings to enhance accountability and transparency in the field. Future studies should involve not only nurses but also physicians, healthcare assistants, patients, and their families to obtain a more comprehensive understanding of current care practices, challenges, and outcomes.

Given the potential for bias in self-reported data, we took several measures to address this concern. During the extended 4-month data collection period, a relevant number of total observations (*n* = 197) were obtained, which may have minimized the impact of social desirability bias. Moreover, we developed a rapport with the ward nurses during this period and conducted unobtrusive observations, where the lead researcher asked questions only when necessary, using neutral language to avoid influencing their responses.

As the main project follows an action-research approach, the identification of current barriers to evidence-informed nursing practice is vital during this initial phase. Previous research has shown that targeted education and training sessions, enhanced supervision and support, protocols, and more effective documentation systems can enhance care outcomes related to CAUTI prevention [24,30].

Although these interventions have been shown to be effective, research has found that adherence rates may decline over time if they are not sustained or if healthcare staff do not perceive changes in structural and procedural dynamics as valuable. Therefore, it is crucial to share these findings with the observed nursing team, foster discussion about current practices, and promote active collaboration in the selection of feasible and effective interventions that suit their specific context and dynamics. Therefore, we will carry out a focus group with the nurses of the department where this study was conducted to identify barriers to evidence-based practice and relevant strategies to be implemented based on these results.

## 5. Conclusions

This study was the first in Portugal to examine nurses’ practices during urinary catheter insertion and maintenance in patients with cancer. Our findings reveal that current nursing practices are not standardized or evidence-based, potentially leading to negative impacts on care outcomes and patient satisfaction. We found significant deviations from international standards of care, particularly in areas such as patient involvement, adherence to aseptic technique during catheter insertion, adherence to hand hygiene and PPE protocols, and inadequate documentation practices.

## Figures and Tables

**Table 1:** Criteria of the Portuguese standard for preventing CAUTIs, along with their sub-criteria and information sources.

Criteria	Sub-Criteria	Data Source
1. Systematically assess whether urinary catheterization can be avoided and document the reasons for urinary characterization in the patient record.	Assess whether urinary catheterization can be avoided.	Nurses’ feedback (Yes/No)
	Document the reasons for urinary catheterization.	
2. Use aseptic technique when inserting the urinary catheter and connecting it to the drainage system.	Use aseptic technique when inserting the urinary catheter.	Observation (Yes/No)
	Use aseptic technique when connecting the urinary catheter to the drainage system.	
3. Use a clean technique, including good hand hygiene and use of gloves and apron, when handling the drainage system of each patient, keeping the urinary catheter connected to the drainage system.	Perform hand hygiene before patient contact.	Observation (Yes/No)
	Perform hand hygiene after the risk of exposure to fluids.	
	Promote appropriate glove usage.	
	Promote appropriate apron usage.	
	Connect the urinary catheter to the drainage system.	
4. Promote daily hygiene of the urethral meatus, by the patients (whenever possible) or the healthcare workers, and provide education to patients and their families about CAUTI prevention.	Promote daily hygiene of the urethral meatus.	Observation and nurses’ feedback (Yes/No)
	Provide patient education about CAUTI prevention.	
5. Secure the urinary catheter, position the drainage bag below the level of the bladder, and empty it when it is 2/3 full.	Secure the urinary catheter.	Observation (Yes/No)
	Position the drainage bag below the level of the bladder.	
	Monitor and empty the drainage bag when it is 2/3 full.	
6. Review the need for the urinary catheter daily, remove it as soon as possible, and document the reasons for keeping it in place in the patient record on a daily basis.	Review the need for the urinary catheter daily.	Nurses’ feedback (Yes/No)
	Document the reasons for keeping the urinary catheter.	

**Table 2:** Nursing practices recorded based on the bundle’s sub-criteria.

Sub-Criteria of Insertion Care (Bundle Criteria 1 and 2)	Episodes with Reliable Data *n*	Overall Adherence *n* (%)
Assess whether urinary catheterization can be avoided	9	9 (100%)
Document the reasons for urinary catheterization	9	7 (77.8%)
Use aseptic technique when inserting the urinary catheter	9	6 (66.7%
Use aseptic technique when connecting the urinary catheter to the drainage system	9	9 (100%)
**Sub-Criteria of Maintenance Care** **(Bundle Criteria 3–6)**		
Perform hand hygiene before patient contact	128	89 (69.6%)
Perform hand hygiene after the risk of exposure to fluids	94	79 (84.0%)
Promote appropriate glove usage	92	86 (93.5%)
Promote appropriate apron usage	89	58 (65.2%)
Connect the catheter to the drainage system	197	194 (98.5%)
Promote daily hygiene of the urethral meatus	185	179 (96.8%)
Provide patient education about CAUTI prevention	137 ^1^	2 (1.5%)
Secure the urinary catheter	197	7 (3.6%)
Position the drainage bag below the level of the bladder	196	185 (94.4%)
Monitor and empty the drainage bag when it is 2/3 full	194	183 (94.3%)
Review the need for the urinary catheter daily	197	197 (100%)
Document the reasons for keeping the urinary catheter in place	197	15 (7.6%)

^1^ Considering only patients without a diagnosis of cognitive impairment.

**Table 3:** Criteria adherence and bundle adherence related to urinary catheter insertion (*n* = 9).

Criteria of Insertion Care	Criteria Adherence	Bundle Adherence
1. Systematically assess whether urinary catheterization can be avoided and document the reasons for urinary catheterization in the patient record	77.8%	55.6%
2. Use aseptic technique when inserting the urinary catheter and connecting it to the drainage system	66.7%	

**Table 4:** Bundle adherence related to urinary catheter maintenance care (*n* = 51).

Criteria of Maintenance Care	Criteria Adherence	Bundle Adherence
3. Use a clean technique, including good hand hygiene and use of gloves and apron, when handling the drainage system of each patient, keeping the urinary catheter connected to the drainage system	47.1%	0%
4. Promote daily hygiene of the urethral meatus, by patients (whenever possible) or healthcare workers, and provide education to patients and their families about CAUTI prevention	0%	
5. Secure the urinary catheter, position the drainage bag below the level of the bladder and empty it when it is 2/3 full	5.9%	
6. Review daily the need for the urinary catheter, remove it as soon as possible, and document the reasons for keeping it in place in the patient record on a daily basis	3.9%	

## Data Availability

The data presented in this study are available on request from the corresponding author.

## Public Involvement Statement

No public involvement in any aspect of this research.

## Guidelines and Standards Statement

This manuscript was drafted against the Strengthening the Reporting of Observational Studies in Epidemiology (STROBE) for cross-sectional studies [14]. A complete list of reporting guidelines can be accessed via the equator network: https://www.equator-network.org/ (accessed on 28 August 2023).
